# Parafascicular Thalamic and Orbitofrontal Cortical Inputs to Striatum Represent States for Goal-Directed Action Selection

**DOI:** 10.3389/fnbeh.2021.655029

**Published:** 2021-03-19

**Authors:** Sandy Stayte, Amolika Dhungana, Bryce Vissel, Laura A. Bradfield

**Affiliations:** ^1^Centre for Neuroscience and Regenerative Medicine, University of Technology Sydney, Sydney, NSW, Australia; ^2^St. Vincent’s Centre for Applied Medical Research, St. Vincent’s Hospital Sydney, Sydney, NSW, Australia

**Keywords:** state, context, goal-directed action, orbitofrontal cortex, parafascicular thalamic nucleus, cholinergic interneurons, striatum

## Abstract

Several lines of evidence accrued over the last 5–10 years have converged to suggest that the parafascicular nucleus of the thalamus and the lateral orbitofrontal cortex each represent or contribute to internal state/context representations that guide action selection in partially observable task situations. In rodents, inactivations of each structure have been found to selectively impair performance in paradigms testing goal-directed action selection, but only when that action selection relies on state representations. Electrophysiological evidence has suggested that each structure achieves this function *via* inputs onto cholinergic interneurons (CINs) in the dorsomedial striatum. Here, we briefly review these studies, then point to anatomical evidence regarding the afferents of each structure and what they suggest about the specific features that each contribute to internal state representations. Finally, we speculate as to whether this role might be achieved interdependently through direct PF→OFC projections, or through the convergence of independent direct orbitofrontal cortex (OFC) and parafascicular nucleus of the thalamus (PF) inputs onto striatal targets.

## Introduction

To select the optimal action in many given situations, it has been posited that organisms must mentally represent that situation (or “internal context,” “latent cause,” or “state;” Gershman et al., 2010; Gershman and Niv, 2012) by integrating features of their internal environment with those of the external environment. To draw upon an example we have given previously (Bradfield and Hart, 2020), if you visit a particular restaurant often, upon visiting you might combine external information about the sights/sounds/smells of the restaurant itself with internal knowledge that it is Saturday and therefore pasta is the daily special, before making an order for pasta. This integrated “state” representation has been claimed to rely on both the parafascicular nucleus of the thalamus (PF) and the orbitofrontal cortex (OFC), particularly when it requires unobservable features to be inferred from memory. How exactly each structure might achieve this function, however, has yet to be considered.

The first suggestion that the PF of the rat might provide information about internal state and/or context to “higher centers” was made in Deschênes et al. (1996) based on the unique morphology of the PF’s glutamatergic projection neurons, the specific topographical arrangement of their outputs to basal ganglia and cerebral cortex, and the rich variety of excitatory and inhibitory afferents PF receives. These features, the authors argued, meant that PF was well-placed to integrate multiple and varied synaptic inputs, and combine them in a way that addressed specific pools of neurons as one might expect of a region combining multiple elements into a unified contextual representation. Twenty-four years later, there have now been several studies employing various techniques, manipulations, and behavioral assays, that have converged to suggest that PF does indeed provide some kind of internal state or context representation (Brown et al., 2010; Bradfield et al., 2013a; Aoki et al., 2015; Bradfield and Balleine, 2017). This information is suspected to influence action selection *via* PF inputs onto cholinergic interneurons (CINs) in the dorsomedial striatum, which is thought to modulate local spiny projection neurons (SPNs), which then co-ordinate to select actions following the currently inferred internal state (Bradfield et al., 2013a; Matamales et al., 2016; Apicella, 2017). Although it is beyond the scope of the current review, recent evidence suggests that how SPNs perform this action selection function is *via* outputs to the substantia nigra reticulata (SNr), through both the direct and indirect pathways. Most recently, it has been suggested that although the direct pathway is always involved in goal-directed action selection, the indirect pathway outputs become particularly important for action selection when unobservable information must be inferred (e.g., during a reversal, Peak et al., 2020).

This role for PF appears to parallel that of the lateral OFC in many ways, at least in rodents (Wilson et al., 2014; Parkes et al., 2018; Bradfield and Hart, 2020). Moreover, the modulation of action selection according to the internal state by dorsomedial CINs has also separately been shown to rely on inputs from lateral OFC (Stalnaker et al., 2016). In this review article, we will explore how the PF and OFC might function independently and/or possibly interdependently to form cohesive representations of the internal state.

### Inactivating the Parafascicular Thalamic Nucleus or Lateral Orbitofrontal Cortex Impairs Goal-Directed Action Selection That Relies on State Representations

Because we and others have extensively reviewed the studies of behavioral consequences of PF and lateral OFC inactivations for goal-directed action elsewhere (Bradfield et al., 2013b; Wilson et al., 2014; Sharpe et al., 2019; Bradfield and Hart, 2020), for present purposes we shall do so only briefly, to reveal their commonalities. More specifically, we have limited our review to studies that employed tasks in which animals could make a goal-directed choice between two or more options that have been studied with regards to both PF and lateral OFC. It is worth noting, however, that numerous other studies (e.g., Brown et al., 2010; Baltz et al., 2018; Malvaez et al., 2019; Zhou et al., 2019) indicate a role for either OFC or PF separately in representing states with regards to other psychological phenomena such as incentive learning and maze learning. It will be of some interest to future studies to employ such tasks in the examination of the alternate structure (i.e., if OFC was studied previously, to study PF using the same task) to determine whether OFC and PF function also appear consistent across those tasks.

The first common finding involving goal-directed choice is that lesions of both PF and lateral OFC have been found to leave instrumental outcome devaluation—the primary behavioral assay used in the laboratory to assess goal-directed action—intact (Ostlund and Balleine, 2007; Balleine et al., 2011; Bradfield et al., 2013a; Bradfield and Balleine, 2017; but see Gremel and Costa, 2013). This suggests that goal-directed action *per se* does not depend on the integrity of either PF or OFC. For the procedure employed in each of these studies, rats were trained to press two levers for two food outcomes (e.g., left-lever pellets, right lever-sucrose, or the opposite arrangement, counterbalanced). Animals were then tested for their ability to flexibly alter their responding in a goal-directed manner when one of the outcomes was reduced in value as a result of it being fed to satiety (i.e., sensory-specific satiety, Balleine and Dickinson, 1992) and animals were subsequently allowed to choose which lever to press. All groups in all of these studies, regardless of whether they had received excitotoxic or sham lesions of PF or lateral OFC, selectively responded on the lever on the test that had previously earned the still-valued outcome. This intact performance suggested that all animals were able to elicit actions motivated by both: (a) the current value of the outcome; and (b) the contingency between action and outcome, and thus fulfilled the two goal-directed action criteria (Balleine and Dickinson, 1998).

Despite this result, there is evidence that another measure of goal-directed action, contingency degradation, is impaired by PF lesions in rats (Bradfield et al., 2013a), as well as by the selective knockdown of brain-derived neurotrophic factor (BDNF) used to reduce activity-dependent neuroplasticity in the lateral OFC of mice (Zimmermann et al., 2017). For contingency degradation, rats are typically once again trained to press the left and the right lever for a pellet and a sucrose outcome, respectively (counterbalanced). After several days of training, one of these outcomes also begins to be delivered freely, in the absence of lever press. This is done in a manner such that the probability of receiving a pellet (if pellets are the degraded outcome) is equivalent regardless of whether the animal presses the pellet lever or not. This serves to degrade the contingency between that specific lever and its outcome, which is evidenced when the animal reduces its pressing on the pellet lever but continues to press the sucrose lever.

**Figure 1:**
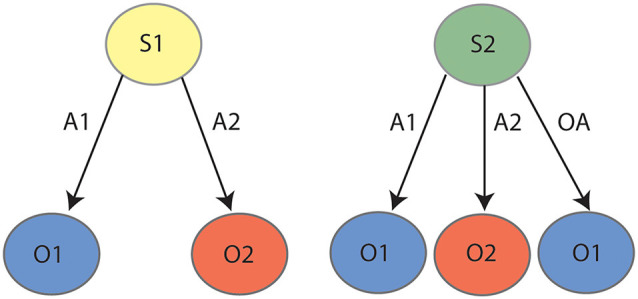
State-space representation for contingency degradation: during the initial phase (left panel) the animal learns that action 1 (A1) earns outcome 1 (O1) and action 2 (A2) earns outcome 2 (O2). During degradation (right panel), the animal learns that in addition to A1–O1 and A2–O2, taking any “other action” (OA, anything other than pressing the levers, e.g., sitting, sniffing, exploring, et cetera) also earns O1, which serves to degrade the contingency between A1 and O1. It is posited that the animal partitions the initial learning contingencies (A1–O1, A2–O2) and the degradation contingencies (A1–O1, A2–O2, OA–O1) into two states, state 1 (S1) and state 2 (S2), respectively.

As reviewed previously (Bradfield and Hart, 2020), successful contingency degradation performance requires animals to reduce interference between competing contingencies in a manner that outcome devaluation does not. Specifically, whereas outcome devaluation requires only that the animal learn only two excitatory lever press-outcome associations (e.g., left lever-pellets, right lever sucrose, represented in the left panel of Figure 1), contingency degradation requires the animal to first learn these same associations, but then to also learn a “no lever press-pellet” association that competes with the left lever-pellet association. We (Bradfield et al., 2013a, b) and others (Schoenbaum et al., 2013) have speculated that in instances such as these, the animal does not unlearn the initial contingency, but rather retains it alongside the new (no lever press-pellet) contingency, and uses internal context/state information to infer whether pressing the pellet lever or abstaining from pressing it is the optimal action to earn a pellet. That is, as shown in Figure 1 and in a manner reminiscent of Yael Niv’s latent cause theory (Gershman et al., 2010; Gershman and Niv, 2012); if the animal infers the initial lever press acquisition state/latent cause [state 1 (S1)] it will press the lever to earn a pellet, but if it infers the degradation state/latent cause [state 2 (S2)] it will withhold lever pressing to earn a pellet. Thus, the fact that PF inactivation and lateral OFC inactivation/BDNF knockdown impairs contingency degradation but not outcome devaluation is consistent with a role for each in representing states, because only degradation requires the partitioning of competing contingencies into separate states.

**Figure 2:**
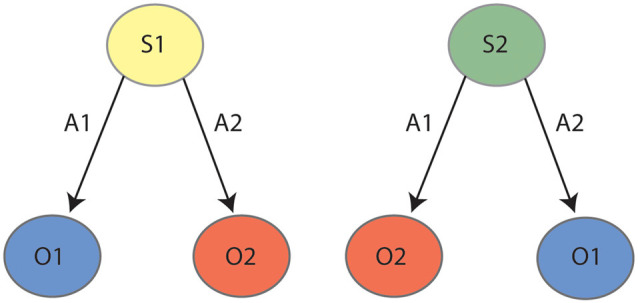
State-space representation for outcome reversal: during the initial phase (left panel) the animal learns that action 1 (A1) earns outcome 1 (O1) and action 2 (A2) earns outcome 2 (O2). During reversal (right panel), the animal learns the reverse contingencies: A1–O2 and A2–O1. It is posited that the animal partitions the initial learning contingencies (A1–O1, A2–O2) and the reversed contingencies (A1–O2, A2–O1) into two states, state 1 (S1) and state 2 (S2), respectively.

A final behavioral assay that is impaired by inactivations of both PF and lateral OFC is reversal learning. As reviewed previously (Manning et al., 2020), the regulation of reversal learning by lateral OFC has been demonstrated across many varied paradigms, but for current purposes, we will describe the only such paradigm that unambiguously recruits goal-directed actions. In this procedure, rats were once again trained to press two levers for two unique outcomes, but these contingencies were later reversed. That is, if the left lever initially earned pellets it was reversed to earn sucrose, and if the right lever initially earned sucrose it was reversed to earn pellets. Animals were again subject to an outcome devaluation test as previously described, and intact animals uniformly responded on the lever that earned the valued outcome following the reversed contingencies. Animals that had experienced PF lesions (Bradfield et al., 2013a) or chemogenetic inactivation of the lateral OFC (Parkes et al., 2018), on the other hand, responded on both levers equally, suggesting that the initially-learned lever press contingencies were interfering with the performance of the reversed contingencies. As shown in Figure 2, it has been posited that animals partition the two sets of contingencies into two internal state representations: State 1 (initial) and State 2 (reversal). Animals that cannot partition the states in this manner would be expected to respond as per both sets of contingencies and press the levers equally, as was observed for animals with PF or lateral OFC inactivation. We have further explicitly demonstrated that intact animals do concurrently retain both the initial and the reversed contingencies in this paradigm (Bradfield and Balleine, 2017), indicating that they must use internal state information to determine with which set of contingencies to act in accordance.

### Parafascicular and Orbitofrontal Cortical Efferents Onto Striatal Cholinergic Interneurons

The behavioral studies reviewed above reveal some striking parallels between the function of the PF and of the lateral OFC in tasks for which goal-directed choices rely on mental representations of states. Additional evidence from electrophysiological, immunohistochemical, and behavioral studies have further demonstrated that this function of PF/OFC inputs manifests *via* inputs onto striatum, particularly (but not exclusively) the cholinergic interneurons (CINs), which are purported to use this information to modulate the local SPNs for accurate action selection (Bradfield et al., 2013b; Stalnaker et al., 2016; Bradfield and Balleine, 2017).

With regards to PF, we (Bradfield et al., 2013a) found that asymmetric lesions of PF and posterior dorsomedial striatum produce identical behavioral results to bilateral PF lesions: leaving outcome devaluation intact but impairing contingency degradation and outcome reversal learning. Interestingly, asymmetric lesions of the PF and *anterior* dorsomedial striatum did not affect behavior in any of our tasks, suggesting that this particular function is specific to the PF→posterior dorsomedial striatal pathway. Further, we demonstrated that lesioning the PF selectively reduced both the action potential frequency, as well as the phosphorylation of ribosomal protein S6 (indicative of reduced CIN activity) of CINs in the dorsomedial striatum, whereas it appeared to *increase* SPN activity in the same region, as evidenced by a significant increase in the number of phospho-Thr202-Tyr204-ERK1/2 (pERK1/2)-labeled SPNs. Thus, although PF does project directly onto SPNs as well as CINs, this particular pattern of responding is most consistent with SPN reactivity being modulated indirectly throughout this task, *via* the loss of CIN modulation as a result of PF lesion, because the direct loss of glutamatergic PF inputs onto SPNs should have manifested as a decrease rather than an increase in SPN activity. Matamales et al. (2016) later directly implicated CINs in dorsomedial striatum in state inference when they demonstrated that the selective toxigenic ablation of CINs in this region also replicated the behavior observed after bilateral PF lesions, leaving outcome devaluation intact but impairing outcome reversal.

With regards to the lateral OFC, Stalnaker et al. (2016) first recorded directly from putative dorsomedial striatal CINs in rats whilst they performed a task in which different outcomes (vanilla or chocolate milk) were earned in different amounts (one or three drops) by different responses (left or right), depending on which “block” was currently active (e.g., in block 1, a left response may have earned three drops of vanilla milk and a right response earned one drop of chocolate milk, et cetera). Switches between blocks occurred in the absence of any change in external stimuli such that rats needed to infer the block change when responses earned different outcomes. Optimal choices following block transition would be more likely if animals were able to infer a new state, rather than overwriting their prior learning about each action-outcome pairing. They found that the activity of CINs was selective to particular blocks and that if this activity appeared to miscode a block, the animal made poorer choices. Moreover, the SPNs recorded in the same study were significantly worse than the CINs at decoding block identity. Finally, when the lateral OFC was lesioned in one hemisphere, CINs in the ipsilateral dorsomedial striatum reduced block decoding to chance level (and interestingly, enhanced decoding of single events). Together, these results suggest that in intact animals, CINs infer some kind of state representation that enables the animal to identify the currently active block, allowing them to make accurate choices accordingly, and they further suggest that this function is dependent on inputs from lateral OFC.

Together, these studies suggest that dorsomedial striatal CINs infer state representations that are used to guide action selection, and that this role for striatal CINs depends on inputs from PF and lateral OFC. It is worth noting, however, that this work is not definitive and almost certainly presents an oversimplified characterization of the mechanisms that underlie the psychological phenomenon of state representation. Indeed, there are complexities both within the striatum (e.g., other neurons/interneuron types), as well as PF and lateral OFC circuitry with other brain regions, that are not captured here that are likely to also contribute to state representation and its influence over action selection.

### Anatomical Evidence for Lateral Orbitofrontal Cortical and/or Parafascicular Representations of Internal State

**Figure 3:**
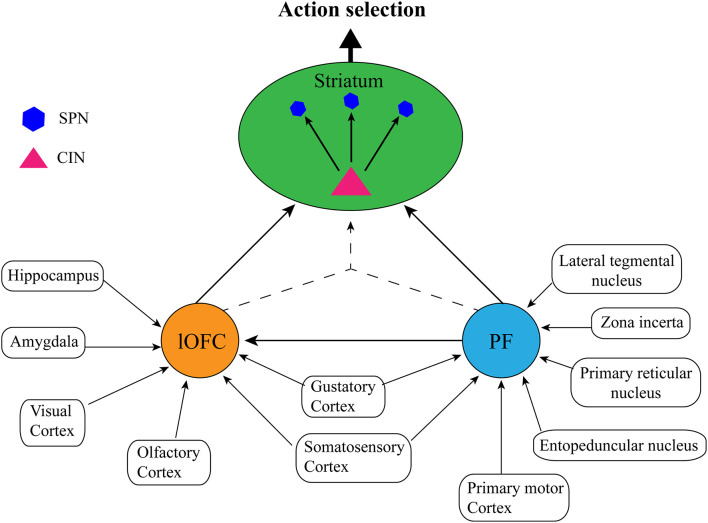
Proposed simplified circuit of internal state representations: The lateral orbitofrontal cortex (lOFC) receives afferents conveying sensory information from visual, olfactory, gustatory, and somatosensory cortices, spatial and memory information from the hippocampus, and emotional information from the amygdala, the parafascicular nucleus of the thalamus (PF) receives sensory inputs from the gustatory cortex and somatosensory cortex, as well as action planning and execution, arousal/attentional information from the lateral tegmental nucleus, zona incerta, primary reticular nucleus, entopeduncular nucleus, and the primary motor cortex. Each structure performs independent integration of these inputs, and then either communicates directly *via* PF→lateral OFC→striatal cholinergic interneurons (CINs), or by converging lateral OFC→striatal CINs/PF→striatal CINs projections to form a cohesive, single internal state representation. Striatal CINs then modulate the striatal projection neurons (SPNs) to influence action selection according to the internal state.

Caveats aside, it is clear from the studies outlined above that PF, OFC, and dorsomedial striatal CINs work in concert with each other (and possibly with other neuronal types/brain regions) to achieve accurate state representation and subsequent goal-directed action selection. What is not clear is how the PF and lateral OFC might coordinate to achieve this function. For instance, do PF and lateral OFC contribute similar or unique information to the state representation? Do they achieve this independently, interdependently, or both? Although it is not possible to answer these questions with any certainty as there have not been any direct studies of how PF-OFC circuitry might relate to internal state representation, here we will provide our speculative view based on the anatomical connections of each structure. Although the characterization of this circuitry presented here has been simplified for ease of communication (see Figure 3), it is worth noting that both OFC (Reep et al., 1996; Hoover and Vertes, 2011) and PF (Mandelbaum et al., 2019) display heterogeneity that is often topographical with regards to their projection patterns which likely underlie different functions. Thus, the state representation function might only rely on the very specific projections that were the focus of the studies above i.e., lateral OFC→centrodorsomedial striatum (Stalnaker et al., 2016; PF→posterior dorsomedial striatum Bradfield et al., 2013a; Bradfield and Balleine, 2017). Nevertheless, there is some suggestion that state representation as it could relate to non-goal-directed, stimulus-dependent responding could rely on adjacent but non-identical neuronal ensembles/pathways (e.g., PF (anterior dorsomedial striatum, dorsolateral CINs; Brown et al., 2010; Aoki et al., 2015, 2018).

As has been noted elsewhere (Schuck et al., 2018; Bradfield and Hart, 2020), the OFC is anatomically well-placed to represent internal state because it receives sensory inputs of multiple modalities, including from olfactory, gustatory, visual, and somatic/sensory cortices. It also receives inputs from regions involved in learning and memory such as the hippocampus, and emotion such as the amygdala. The lateral OFC is thus well-placed to integrate information about an organism’s current circumstances from multiple sensory inputs with learned information from memory about which actions may have historically resulted in optimal outcomes in the current environment, as well as emotional information about the organism’s current desires. This is precisely the type of information that is necessary to infer internal states for accurate action selection in partially observable task situations.

As mentioned previously, the PF has also been noted for being anatomically well-placed to receive and integrate several disparate inputs in the way one might expect of a structure encoding internal state or context information (Deschênes et al., 1996). Similar to lateral OFC, the rat PF receives some sensory inputs that could allow it to identify current circumstances, such as inputs from the gustatory cortex and inputs from the primary somatosensory cortex (Cornwall and Phillipson, 1988). In contrast to lateral OFC, however, PF does not receive any direct inputs (that we know of) from visual, auditory, or olfactory cortices, hippocampus, or amygdala. In further contrast to the lateral OFC, all other primary sources of inputs to PF appear to play some role in motor function or arousal/attention. Specifically, major inputs to PF include those from the primary motor cortex, primary reticular nucleus, entopeduncular nucleus, zona incerta, and laterodorsal tegmental nucleus (Cornwall and Phillipson, 1988), each of which plays some role in planning and executing movements, and/or mediating attention and arousal. Although we will not address it further here, the mediation of attention, in particular, has also been persuasively argued to be central to PF’s contextual regulation of dorsomedial striatal CINs (see Apicella, 2017).

Overall, therefore, it would appear that the afferents of the lateral OFC and PF are relatively distinct, despite the apparent similarity of these structures concerning their cognitive/behavioral function regarding goal-directed choices. In our view, the information regarding motor responses that are conveyed to PF could be the missing puzzle piece that, in addition to the sensory, emotional, and memory-based inputs received by lateral OFC, is necessary for the formation of a single, cohesive state representation. This can be illustrated using the example of the goal-directed reversal learning procedure outlined above. As mentioned, we (Bradfield and Balleine, 2017) have previously demonstrated that animals who have undergone reversal learning simultaneously retain both sets of competing contingencies, for example, State 1: left lever-pellets, right lever sucrose; State 2: left lever-sucrose, right lever pellets (see Figure 2). To effectively form and distinguish between these state representations in the absence of any change in context or stimuli, it seems that motor information about which action is being/has been performed would be crucial to link it with which outcome is ultimately earned. For example, if on one day after placement in the operant chamber the animal presses the left lever and earns pellets, for example, it might infer state 1, whereas on another day it might press the left lever and earn sucrose, thus inferring state 2. Such motor information about which lever is pressed appears to be readily available *via* multiple inputs to the PF but is not available to lateral OFC.

If our assertion here is correct, it assumes that the lateral OFC and PF both integrate disparate information which is then further integrated to form a single, internal state representation to influence action selection. How this could work in a practical sense is illustrated by the following example: when the animal is initially placed into the operant chamber, any state inference that it makes initially would be based on the integration of their current sensory inputs (telling them they are in the operant chamber) combined with their memory of what happened in the operant chamber the day before (e.g., yesterday State 1 was active so infer State 1 will be active again today). Based on the afferents outlined above, this initial inference is most likely to rely on lateral OFC. However, it is only when the left lever is actually pressed and pellets are delivered to the food receptacle that the animal can confirm that State 1 and not State 2 is currently active, and this inference more likely relies on PF that receives feedback about the motor action performed. In a broader sense, we, therefore, suggest that whereas the lateral OFC might form integrated representations about the sights and smells of the environment with memories about what contingencies were previously active here and what outcome(s) is currently desirable, whereas the PF might form and infer integrated representations of current sensory circumstances with motor response-outcome information. One straightforward way in which to test whether this is the case would be to demonstrate that performance in a task that does not require prior motor response-outcome history for state representation formation does not rely on PF. An example of such a task would be sensory preconditioning, which involves the learning of “value-free” stimulus-stimulus associations and has been shown to relate to neuronal activity in the lateral OFC (Sadacca et al., 2018), consistent with lateral OFC forming state representations based on memories, emotions, and external information in the absence of motor history information.

If the OFC and PF do function in the manner described here, one final question is whether they each form their own, unique but partial state representations which individually influence striatal CINs, or whether they contribute unique information to a single state representation which either influences CINs or is formed by the striatal CINs themselves. What is clear from the inactivation studies outlined above, and suggested by the afferents of each structure, is that both the PF and lateral OFC do form some kind of integrated representation rather than just representing individual elements of state representation. If either structure did the latter (e.g., impaired motor learning generally) we would expect their inactivation to produce broader behavioral deficits in learning and performance, not the specific deficits that were only observed once contingencies were altered.

There is a direct projection from PF to lateral OFC (Reep et al., 1996; Hoover and Vertes, 2011) suggesting a potential anatomical basis for these structures to communicate to form a cohesive representation of the internal state. This is not a particularly dense pathway, however, and there is no evidence of any reciprocal projections from OFC→PF, suggesting that any communication between them must be unidirectional. Thus, if a cohesive state representation were formed *via* this pathway alone, then the PF could only influence striatum indirectly *via* lateral OFC→striatal outputs. Given the density of the direct pathway from PF→dorsomedial striatum, this seems unlikely. An alternate possibility is that OFC inputs containing a unique sensory/emotion/memory representation, and PF inputs containing a unique sensory/motor representation, converge on the same striatal CINs, which then combine postsynaptically to form a cohesive state representation. This possibility is more consistent with our prior notion that internal state information is formed postsynaptically by CINs (Bradfield et al., 2013a). A final possibility is that both of these things occur: the PF projects to both OFC and striatal CINs and OFC also projects to striatal CINs, to achieve accurate goal-directed selection according to the currently inferred state.

### Conclusion and Potential Implications

In summary, behavioral evidence from local inactivation studies suggests that there are certain parallels between the function of the lateral OFC and PF with regards to the modulation of goal-directed actions involving choice. Specifically, the patterns of results yielded from these studies are suggestive of each structure forming some kind of internal state representation that is then used to guide goal-directed actions (although this role for lateral OFC may also extend to “model-free” habitual actions: see Wilson et al., 2014). Here, we have briefly reviewed these parallels, before discussing anatomical evidence that may be suggestive of how the OFC and PF could complement each other in achieving this function. As these observations are purely speculative at this stage, it will be of interest to determine whether they are borne out by future studies.

If correct, and translatable between species, this function of PF/OFC/CINs could be of great importance. There is already some suggestion that these functions do translate, based on several lines of evidence suggesting that OFC (Fellows, 2003; Valentin et al., 2007; Balleine and O’Doherty, 2010; Wallis, 2012), PF (Bell et al., 2018a), and striatal CINs (Bell et al., 2018b, 2019) play similar roles in regulating behavioral flexibility in humans to that which has been identified in rodents. In the vastly more rich and complex world that is inhabited by people relative to laboratory rodents, one could imagine that the ability to infer internal states accurately to exercise the appropriate action to each situation could be central to effective functioning. Every time one drives a different car, for example, they would have to infer the slight differences in how hard to press the accelerator or brakes, or whether they are driving manual or automatic and thus need to change gears, all whilst paying attention to the road. If this ability is lost, as it potentially is in Parkinson’s disease patients who experience not only a loss of dopaminergic inputs to striatum but also a loss of thalamic inputs to striatal CINs (Smith et al., 2014, 2016), it could deeply impair the ability of individuals to flexibly switch between actions as each situation dictates. This is potentially the neurobiological mechanism that underlies “cognitive rigidity” that is typical of individuals with Parkinson’s disease dementia (Kehagia et al., 2010; Smith et al., 2016).

Despite some functional similarities across species, however, there is some anatomical evidence to suggest that the nature of PF projections to striatum and cortex does differ somewhat between rodents and primates. Specifically, it has been noted that, in rats, it is the same PF neurons that project directly to the striatum that also project to cortical regions (Deschênes et al., 1996), but this was found not to be the case in primates, where striatal-projecting neurons and cortical-projection neurons in PF appear to be separate (Parent and Parent, 2005). Thus, although there is evidence of some parallels across species with regards to the function of the PF and OFC inputs onto CINs within the striatum, there are also likely differences in how this manifests across species. Again, future studies are necessary to determine how much anatomical, functional, and cytoarchitectural homology there is between these structures and circuits in rodents, primates, and humans, and how applicable these findings could be to individuals with various conditions affecting them such as Parkinson’s disease dementia.

## Author Contributions

LB completed the first draft. AD, SS, and BV contributed to and edited this draft. SS and LB created the figures. All authors contributed to the article and approved the submitted version.

## Conflict of Interest

The authors declare that the research was conducted in the absence of any commercial or financial relationships that could be construed as a potential conflict of interest.
